# Proteomics Computational Analyses Suggest That the Envelope Glycoproteins of Segmented Jingmen Flavi-Like Viruses are Class II Viral Fusion Proteins (β-Penetrenes) with Mucin-Like Domains

**DOI:** 10.3390/v12030260

**Published:** 2020-02-27

**Authors:** Courtney E. Garry, Robert F. Garry

**Affiliations:** 1 School of Nursing, Johns Hopkins University, Baltimore, MD 21205, USA; courtneygarry@gmail.com; 2 Department of Microbiology and Immunology, Tulane University School of Medicine, New Orleans, LA 70112, USA; 3 Zalgen Labs, Germantown, MD 20876, USA

**Keywords:** Jingmen tick virus, Alongshan virus, flavi-like viruses, class II viral fusion proteins, O-liked glycans, mucin-like domain, virus evolution

## Abstract

Jingmen viruses are newly described segmented flavi-like viruses that have a worldwide distribution in ticks and have been associated with febrile illnesses in humans. Computational analyses were used to predict that Jingmen flavi-like virus glycoproteins have structural features of class II viral fusion proteins, including an ectodomain consisting of beta-sheets and short alpha-helices, a fusion peptide with interfacial hydrophobicity and a three-domain architecture. Jingmen flavi-like virus glycoproteins have a sequence enriched in serine, threonine, and proline at the amino terminus, which is a feature of mucin-like domains. Several of the serines and threonines are predicted be modified by the addition of O-linked glycans. Some of the glycoproteins are predicted to have an additional mucin-like domain located prior to the transmembrane anchor, whereas others are predicted to have a stem consisting of two alpha-helices. The flavivirus envelope protein and Jingmen flavi-virus glycoproteins may have diverged from a common class II precursor glycoprotein with a mucin-like domain or domains acquired after divergence.

## 1. Introduction

The *Flaviviridae* family contains several important human and animal pathogens, including dengue, yellow fever, West Nile, hepatitis C, Zika, and bovine viral diarrhea viruses. The family is currently divided into four genera, *Flavivirus*, *Hepacivirus*, *Pestivirus*, and *Pegivirus* [1]. Members of the *Flavivirus* genus are capable of replicating in both insect and vertebrate hosts [2,3]. Hepaciviruses, pestiviruses, and pegiviruses were each considered until recently to be exclusively viruses of mammals [4,5]. Despite extensive genetic divergence, all current members of the *Flaviviridae* have an unsegmented, single-stranded, positive-sense RNA genome of less than 13 kb. The genome is translated into a single polyprotein that is cleaved by host and viral proteases into structural and nonstructural (NS) proteins. The genes for the structural proteins, including a capsid protein C and two glycoproteins, are encoded at the 5′ end of the genome, whereas the genes for the NS proteins are 3′. The genes for NS2B-NS3 (protease/helicase) and NS5 (methyltransferase /RNA-dependent RNA polymerase) are the most conserved amongst the family [4].

Studies conducted over the past several years have demonstrated that viruses with notable similarities to members of the *Flaviviridae* infect a wide range of hosts and exhibit great diversity in genome structure [4,6,7]. In a remarkable metatranscriptomic study Shi, Lin, Vasilakis, Tian et al. retrieved complete genomes of 12 distant *Flaviviridae* virus relatives (“flavi-like” viruses) from a range of invertebrate species, including flies, crickets, centipedes, spiders, and barnacles [4]. Although these viruses diverge from known flaviruses and have genomes that are larger (16–26 kb), they have a similar overall genome organization, polyprotein expression strategy and significance sequence similarities in the NS2B-NS3 and NS5 genes. The wide diversity of flavi-like viruses in invertebrates, coupled with their deep phylogenetic positions, lead the authors to hypothesize that insect flavi-like viruses may represent the ancestral forms from which vertebrate-infecting flaviviruses evolved.

Additional flavi-like viruses have been discovered. These include the Gentian Kobu-sho-associated virus (GKaV) from plants of the genus *Gentiana*, which are a source of aperitif bitters [6]. Flavi-like viruses have also been found in fruit flies [8], lice [9], aphids [10], and a nematode [5]. They have long genomes, a flavi-like genome organization, and sequence similarities to flavivirus NS3 and NS5 genes. In addition, a virus from *Proscyllium habereri*, the graceful catshark, has significant sequence and genome structure similarities to hepaciviruses [4]. The isolation of a close relative of hepaciviruses from a fish suggests that these viruses may possess a broader host range and more ancient evolutionary history than was known previously.

An extreme variation on the genomic structures of flavi-like viruses was revealed with the observation of tick-borne viruses with genomes comprised of four segments. Qin, Shi, Tian, Lin et al. [7] detected Jingmen tick virus (JMTV) in *Rhipicephalus microplus* ticks in Hubei Province, China. Common 5′ and 3′ untranslated region (UTR) nucleotide sequences, including the termini, were present in all four segments, confirming that the JMTV genome is segmented. Segment 1 encodes a protein that has canonical motifs for methyltransferase and RNA-dependent RNA polymerase found in NS5 of unsegmented flaviviruses. Segment 3 encodes a protein with similarities to the flaviviral serine protease domain and helicase domains of NS3 are well as NS2B. Segments 2 and 4 appear to encode the glycoproteins and capsid of JMTV, respectively, but sequence homology searches (BLASTx and BLASTp) found no similarities to any flavivirus proteins [7]. However, homology searches found matches of JMTV segments to the transcripts of a larval cDNA library of *Toxocara canis*, a helminth parasite of dogs and other canids. The four RNAs identified belong to a virus related to JMTV, which was named *T. canis* larva agent (TCLA).

JMTV-related flavi-like viruses are now known to be widespread in ticks worldwide and some have been associated with febrile illness in humans [11,12,13,14]. A retrospective study reported the identification of JMTV in *Amblyomma javanense* and skin biopsies and the blood of febrile patients in China [15]. JMTV has also been isolated from ticks, animals or humans in Kosovo [16], Trinidad and Tobago [17], Turkey [11] and the French Antilles, France, Lao People’s Democratic Republic, and Cambodia [13]. Isolations of other JMTV-related viruses include Mogiana tick virus (MGTV) from *R. microplus* and cattle in Brazil [18,19], Kindia tick virus (KNTV) from *R. geigyi* in Guinea [20] and Yanggou tick virus (YGTV) from *Dermacentor nuttalli* in China [21]. Guaico Culex virus (GCXV) was isolated from a red colobus monkey in Uganda, which contains four segments with sequence similarity to the four segments of JMTV and an apparently optional fifth segment [22]. Alongshan virus (ALSV) was detected in *Ixodes persulcatus* and isolated from febrile patient sera in Heilongjian Province in China [14]. ALSV is also present in *Ixodes ricinus* ticks in the Kotka archipelago of Southeastern Finland [23]. We refer to these segmented flavi-like viruses as Jingmen flavi-like viruses.

In addition to the nonsegmented flavi-like invertebrate viruses, Shi, Lin, Vasilakis, Tian et al. [4] discovered five novel segmented flavi-like viruses from fleas, aphids, crickets, and other insects. Because they are genetically related to each other, we refer to these viruses as Wuhan flavi-like viruses after the type species, Wuhan cricket virus (WHCV). The genome of Wuhan flavi-like viruses consists of four segments, with a gene organization like that of Jingmen flavi-like viruses. Wuhan flavi-like viruses lack the poly(A) tail present at the end of each Jingmen flavi-like virus segment. Like Jingmen flavi-like viruses, segments 1 and 3 of Wuhan flavi-like viruses share sequence similarities with the NS2B-NS3 and NS5 genes of flaviviruses. Despite genetic relatedness and other similarities, the International Committee on the Taxonomy of Viruses (ICTV) has not included flavi-like viruses in the *Flaviviridae* [1]. The non-segmented divergent flavi-like viruses of arthropods, and the segmented Jingmen and Wuhan group viruses are not formally classified. Jingmen and Wuhan flavi-like viruses could potentially be assigned to a *Jingmenivirus* genus within the *Flaviviridae*, or they could be given a separate classification.

Analysis of the glycoproteins encoded by Segment 2 of the segmented Jingmen and Wuhan flavi-like viruses did not reveal similarity to any flavivirus glycoprotein or other viral glyoprotein detectable by commonly used global search programs, such as BLAST [4,7]. Here, we examine the amino acid sequences of proteins encoded by the segmented flavi-like viruses using computational tools that have previously proven potent in identifying structural features of viral fusion proteins [24,25,26,27,28,29].

## 2. Materials and Methods

### 2.1. Sequences

GenBank accession numbers of the segmented flavi-like viruses used for sequence and protein structural analyses are provided in Table 1. Comparisons are also shown to yellow fever virus proteins (strain 17D, GenBank: X03700.1).

### 2.2. Proteomics Computational Methods

Proteomics computational analyses were performed using methods originally developed by William Gallaher [25]. Pairwise alignments were performed using William Pearson’s Lalign program [30] that implements the algorithm of Huang and Miller [31]. To generate alignments between multiple sequences we used Clustal Omega, a part of the EMBL-EBI search and sequence analysis tool kit that uses seeded guide trees and Hidden Markov Model (HMM) profile–profile techniques [32]. Secondary structure, solvent accessibility, transmembrane helices, globular regions, coiled-coil regions, and other protein features were analyzed using PredictProtein [33]. Domains with a significant propensity to form transmembrane helices were identified with TMpred [34]. TMpred is based on a statistical analysis of TMbase, a database of naturally occurring transmembrane glycoproteins [35]. Topology and other features of membrane proteins were explored by means of hydropathy plots with Membrane Protein e**X**plorer (MPeX) from the Stephen White laboratory [36]. MPeX is based on the experimentally determined Wimley–White interfacial hydrophobicity scale (WWIHS) [37,38]. The presence of signal peptides and the location of their cleavage sites was analyzed using Signalp (v5.0), which is based on deep convolutional and recurrent neural network architecture, including a conditional random field [39]. O-linked glycosylation sites were predicted by Net-O-Glyc v. 4.0 [40]. The NetOglyc server produces neural network predictions of mucin type GalNAc O-glycosylation sites.

## 3. Results

### 3.1. Sequence Similarities between Flavivirus and Flavi-Like Virus Proteins

**Figure 1:**
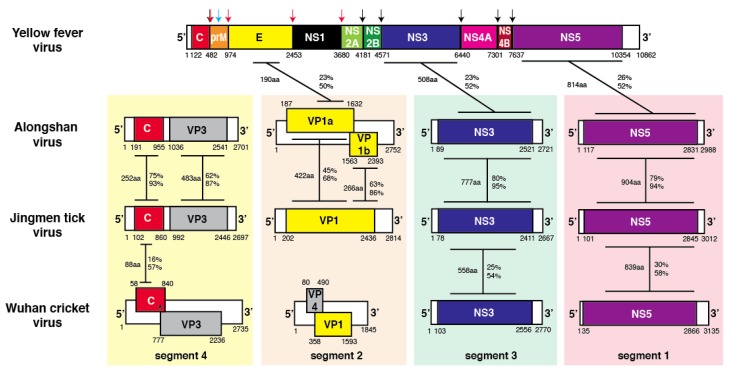
Genomic structure of representative Jingmen and Wuhan flavi-like viruses and yellow fever virus, a member of the *Flaviviridae*. The regions of amino acid similarity between protein pairs are indicated. The numbers of amino acids (aa) and the percent of identical (top) and chemically similar (bottom) amino acids in the overlap is indicated. Arrows indicate proteolytic cleavages of the yellow fever virus polyprotein.

JMTV is a novel tick-borne segmented RNA virus containing genome segments derived from unsegmented viral ancestors [7]. Additional segmented flavi-like viruses with genetic similarity to JMTV (Jingmen flavi-like viruses) have subsequently been isolated from ticks and other invertebrates [12,13]. Another group of novel genetically-related segmented flavi-like viruses has been discovered in insects (Wuhan flavi-like viruses) [4]. The gene order of nonsegmented flaviviruses of the *Flavivirus* genus is 5′ capsid-PrM-envelope-NS1-NS2A-NS2B-NS3-NS4A-NS4B-NS5-3′ (Figure 1). Segment 4 of Jingmen and Wuhan flavi-like viruses encode a capsid protein and a membrane protein. No sequence similarity can be detected between the capsid proteins of segmented or nonsegmented flaviviruses. The capsid proteins of Jingmen flavi-like viruses ALSV and JMTV share substantial amino acid similarities (75% identical and 93% similar amino acids over a 252 amino acid segment). There is limited similarity of the capsid proteins of ALSV and JMTV to the capsid of WHCV (16% identical and 57% similar amino acids over an 88 amino acid segment), but not the membrane protein. There is no apparent close homolog of the membrane protein of the flavi-like viruses in nonsegmented flaviviruses. However, M proteins of flavi-like viruses have several TM domains (10 for ALSV). The membrane proteins of ALSV and JMTV are similar to each other, but not closely related WHCV M.

Segment 2 of segmented flavi-like viruses encode the envelope proteins (Figure 1). Two patterns are noted in Jingmen flavi-like viruses. Segment 2 of ALSV, YGTV, and some other Jingmen flavi-like viruses encode two proteins referred to here as VP1a and VP1b (also known as G1 and G2). Segment 2 of JMTV and MGTV are representative of the majority of known Jingmen flavi-like viruses that encode a single glycoprotein VP1. No apparent similarity of any Jingmen flavi-like virus glycoprotein to glycoproteins of any member of the *Flaviviridae* has been detected previously by BLASTx, BLAST p or other common homology search programs [7]. Jingmen flavi-like viruses displayed limited local alignments with the envelope glycoproteins (E) of nonsegmented flaviviruses. For example, ALSV VP1a shares 23% identical amino acids and 50% chemically similar amino acids with yellow fever virus (YFV) envelope (E) protein over a 190 amino acid length segment (Figure 1). The glycoproteins of the two subgroups of Jingmen flavi-like viruses also share similarities. ALSV VP1a and JMTV VP1 share 45% identical amino acids and 68% chemically similar amino acids over a 422 amino acid length segment beginning near the amino-termini (N-termini) of the proteins ALSV VP1b shares similarities to the carboxy-termini (C-termini) of JMTV VP1. The proteins encoded by segment 2 of Jingmen flavi-like viruses have no sequence similarities to Segment 2 proteins of Wuhan flavi-like viruses. Wuhan flavi-like viruses have a shorter Segment 2 that contains two overlapping open reading frames (ORFs) [4]. The protein products of both Wuhan flavi-like virus ORFs are predicted to have N-terminal signal peptides. The protein encoded by the second ORF contains a C-terminal transmembrane (TM) domain.

As previously noted [4,7,23], Segment 1 and 3 sequences of Jingmen and Wuhan flavi-like viruses encode proteins with significant sequence similarities to the NS3 and NS5 proteins of flaviviruses, respectively (Figure 1). There was limited local similarity between YFV NS2B and the proteins encoded by Segment 3 of ALSV and JMTV. ALSV and JMTV NS3 share 80% identical amino acids and 95% chemically similar amino acids over a 777 amino acid length segment. ALSV and JMTV NS5 share 79% identical amino acids and 94% chemically similar amino acids over a 904 amino acid length segment. Analyses of Segment 1 and 3 support subgrouping JMTV, MGTV, and other viruses encoding a single glycoprotein in a separate subgroup from ALSV, YGTV, and other Jingmen flavi-like viruses that encode two glycoproteins (VP1a and VP1b). JMTV and MGTV NS3 and NS5 proteins are more similar than they are to ALSV NS3 and NS5. JMTV and MGTV NS3 share 97% identical and 99% similar amino acids over an 808 amino acid overlap, while JMTV and MGTV NS5 proteins share 96% identical and 99% similar amino acids over a 914 amino acid overlap. Proteins encoded by Segment 1 and 3 sequences of Jingmen and Wuhan flavi-like viruses are also related. Interestingly, NS3 and NS5 genes of nonsegmented members of the *Flavivirus* genus are more closely related to Jingmen and Wuhan flavi-like virus NS3 and NS5 than to NS3 and NS5 of members of other genuses of the *Flaviviridae* [4].

### 3.2. Sequence Similarities of Jingmen Flavi-Like Virus Glycoproteins

**Figure 2:**
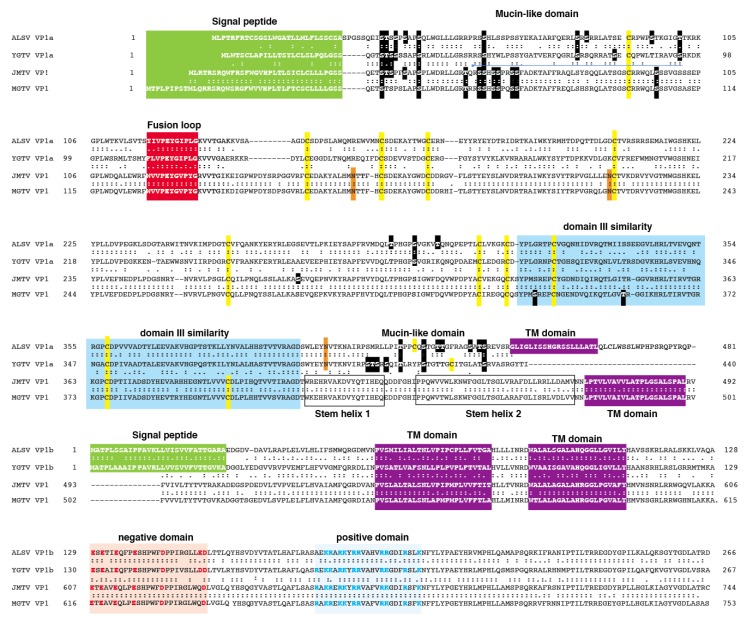
Amino acid sequence alignment of Jingmen flavi-like virus glycoproteins. Amino acid sequences of the glycoproteins from Alongshan virus (ALSV), Yanggou tick virus (YGTV), Jingmen tick virus (JMTV), and Mogiana tick virus (MGTV) are aligned. Yellow highlights: cysteines. Orange highlights: N-glycosylation sites. Black highlights: O- glycosylation sites.

The VP1 of JMTV and MGTV are of similar length, 744 and 753 amino acids, respectively (Figure 2). The two Segment 2 glycoproteins, VP1a and VP1b, of ALSV are 481 and 266 amino acids, respectively. VP1a and VP1b of YGTV are 440 and 267 amino acids, respectively. The VP1, VP1a, and VP1b proteins of Jingmen flavi-like viruses have predicted N-terminal signal peptides of 26 to 42 amino acids. JMTV and MGTV VP1 have three predicted TM domains. Prior to the first transmembrae (TM) domain in JMTV and MGTV are two α-helices predicted by PredictProtein, an advanced secondary structure prediction algorithm. We refer to this region as the stem domain by analogy to the extended α-helices that are present prior to the TM anchoring domains in the E proteins of members of the *Flavivirus* genus [41,42,43] and glycoproteins of other viruses [44]. The VP1a of ALSV has a C-terminal TM domain, but VP1a of YGTV does not. Distinct isolates of ALSV and YGTV have the same predicted structures, so this difference in the presence of C-terminal anchors does not appear to be due to sequencing errors. The C-termini of Jingmen flavi-like virus VP1 and VP1b are similar with two TM domains (for a total of three in VP1). VP1 and VP1b contain a segment enriched in negatively charged amino acids followed by a segment enriched in positively charged amino acids.

### 3.3. Jingmen Flavi-Like Virus Glycoproteins Contain Predicted Mucin-Like Domains

Inspection of the amino acid sequences of the VP1 or VP1a proteins of Jingmen flavi-like viruses revealed several stretches that contain a concentration of proline, serine, and threonine (PST), features that have been associated with mucin-like domains on viral glycoproteins and other glycoproteins (Figure 2) [45,46]. Net-O-Gly predicts that many of these serines and threonines are modified by the addition of an O-glycan. Each of the Jingmen flavi-like viruses have an extended patch of predicted O-glycans at the N terminus of the protein following the predicted signal peptide, which we refer to as a mucin-like domain. Jingmen flavi-like viruses also have one to three additional predicted O-glucan between amino acids 230 and 360. The VP1a of ALSV and YGTV and other flavi-like viruses that encode two glycoproteins are distinguished from JMTV and MGTV and other Jingmen flavi-like viruses that encode one glycoprotein in the presence of the C-terminal domain, which we refer to as the leash by analogy to the pre-anchor domains of certain other VFP [28,47]. The leash is enriched in PST and is predicted to contain seven O-linked glycans constituting another potential mucin-like domain.

### 3.4. Jingmen Flavi-Like Virus Glycoproteins Have Structure Features of Class II Viral Fusion Proteins

A fusion peptide or loop is present in all members of each known class of viral fusion protein [48,49]. These amino acid sequences interact directly with cellular membranes during fusion with the virion membrane [50]. Fusion peptides or loops have the potential to disrupt and partition into bilayer membranes, as revealed by analyses using the Wimley–White interfacial hydrophobicity scale (WWIHS) [37,38]. Fusion peptides or loops are typically among the most highly conserved sequences. Several sequences in Jingmen flavi-like virus VP1 or VP1a proteins fit the canonical profile of a fusion peptide of a class II VFP and produce a positive WWIHS score. Based on location, amino acid composition, and sequence conservation, it is likely that amino acids 119 to 129 in ALSV VP1a and similar sequences in other Jingmen virus glycoproteins represent the fusion peptides (Figure 2).

Class I or III VFPs have two or one α-helices prior to the TM anchoring domains [25,28,51,52,53]. PredictProtein indicates that the Jingmen flavi-like virus glycoproteins do not have a propensity to form extended α-helices, except for the TM domains and the stem helices when present. This suggests that the glycoproteins of Jingmen flavi-like virus do not encode class I or III VFPs. Protein Predict indicates that the ectodomains of Jingmen flavi-virus like glycoproteins have a propensity to form a mixture of β-sheets and short α-helices. This pattern is reminiscent of class II VFPs [27,54,55], which led us to perform pairwise alignments and visual inspection that can detect the limited similarities with flavivirus E not found in large-scale similarity searches (Figure 1). The ectodomains of flavivirus E have three domains. Domain I is in the center of the molecular and can contain a hinge. Domain II contains the fusion peptide and domain III the receptor binding domain. ALSV VP1a has an extended similarity to YFV E domain III (Figure 2). The region contains a pair of conserved cysteines. PredictProtein demonstrates that this region in Jingmen flavi-like viruses has the propensity to form several β-sheets, which is consistent with a domain III structure.

### 3.5. Jingmen Flavi-Like Virus Glycoprotein Models

**Figure 3:**
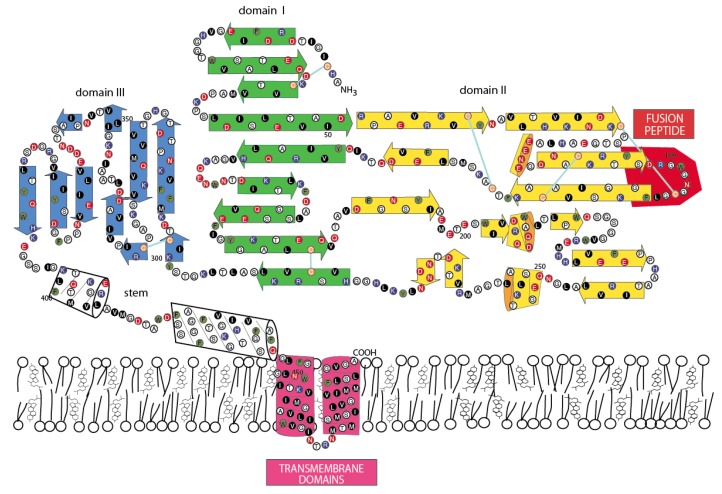
Two-dimensional model of the yellow fever virus envelope glycoprotein E. N-glycosylation sites: orange stick figures: O-linked glycosylation sites: black figures.

**Figure 4:**
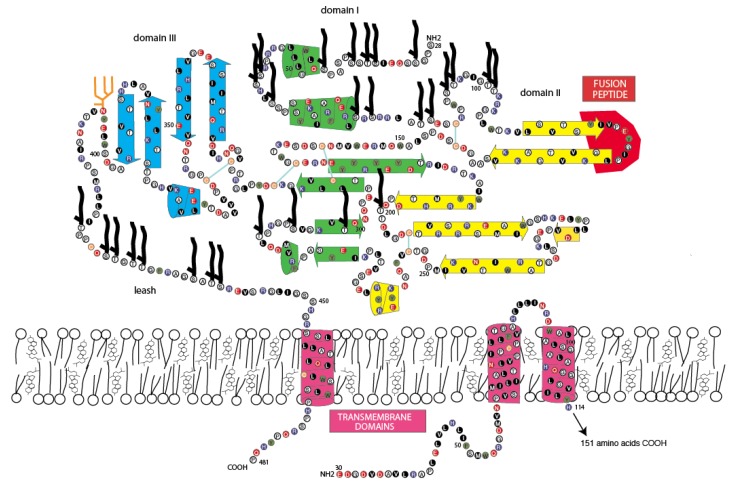
Two-dimensional model of Alongshan virus viral proteins 1a and Ib. N-glycosylation sites: orange stick figures: O-linked glycosylation sites: black figures.

**Figure 5:**
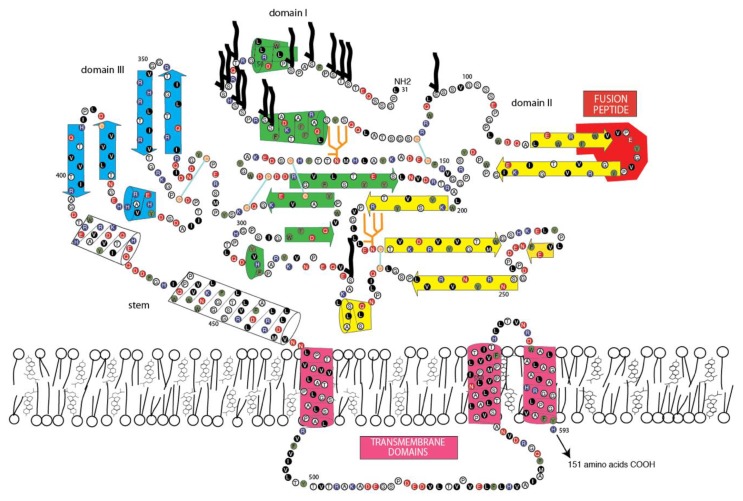
Two-dimensional model of Jingmen tick virus viral protein 1. N-glycosylation sites: orange stick figures: O-linked glycosylation sites: black figures.

To determine whether or not the amino acid sequence of Jingmen flavi-like viruses can plausibly fit a class II VFP architecture similar to the envelope glycoproteins (E) of nonsegmented flaviviruses, we constructed space-filling models. The folding of the glycoprotein must accommodate the location of structure features of the protein and cysteine bonding. Cysteines are usually the most conserved amino acids within a protein family because disulfide linkages are a critical determinant of secondary structure. With the exception of the last cysteine in each of the Jingmen flavi-like virus glycoproteins the cysteines are conserved (Figure 2). Overall, the length and co-linearity of putative fusion loops, class III domains, stem domains (when present) and TM anchors are consistent with the suggestion that Jingmen flavi-like viruses encode modified class II fusion proteins. There are many possible alternatives to the cysteine linkages and secondary structures shown. However, plausible models that conform to the scaffold of the known structure class II VFP can be constructed based on the Jingmen flavi-like virus sequences (Figure 3, Figure 4 and Figure 5). After scaffolding on YFV E structure (Figure 3), the models for ALSV VP1a (Figure 4) and JMTV VP1 (Figure 5) place predicted structures such as the fusion peptides in appropriate domains. The models also place cysteine residues in close proximity with possible linkages occurring within the three domains, while conforming to the structural predictions of PredictProtein.

**Figure 6:**
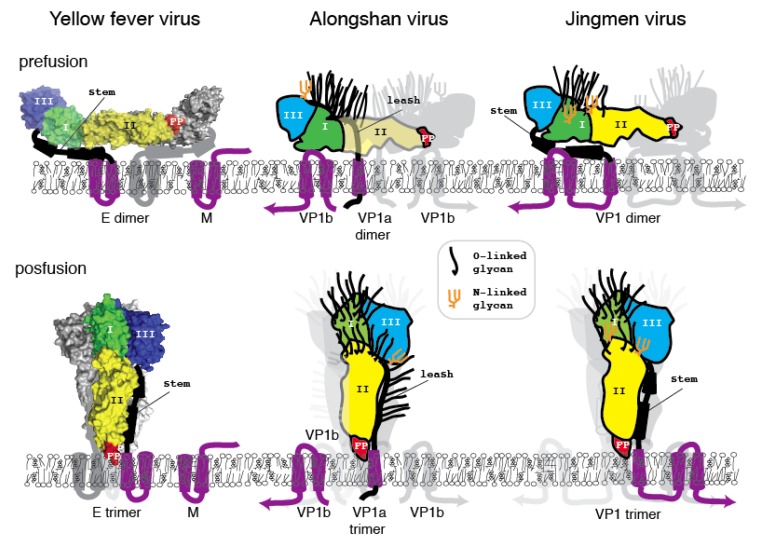
Space filling models of yellow fever virus E, Alongshan virus viral protein 1a and Ib, and Jingmen tick virus viral protein 1. Top: prefusion (virion) forms. Bottom: post-fusion forms.

Class II fusion proteins of members of the *Flavivirus* genus are present on the surface of mature virions as homodimers. Current models of fusion class II viral fusion proteins suggest that exposure to the low pH of the endosome triggers trimerization and a bending of class II fusion proteins at a flexible “hinge” region between domains I and II, elevating the fusion peptide so that it can insert into the host membrane [56,57]. A rearrangement of the stem (pre-anchor region), so that there are more extensive interactions with domains I-III, results in a deformation of the viral and target membranes. Subsequently, the membranes are brought closer together by continued interactions of the stem with domains I-III, which results in bilayer hemifusion [58,59]. The formation of the final post-fusion trimer accompanies complete fusion of the viral and cellular membrane allowing entry of the ribonucleoproteins containing the viral genomic RNA. To examine whether the class II structure of the Jingmen flavi-like viruses could accommodate similar rearrangements, we constructed space-filling models of Jingmen flavi-like virus glycoproteins in dimer and trimer configurations (Figure 6). Mucin-like domains would be exposed on the surface of the proteins. The N-terminal mucin-like domain of both ALSV and JMTV potentially sits on top of the dimer. The mucin-like domain found on the leash of ALSV would be able to occupy the space between the dimers on the virion. The rearrangement of ALSV from a dimer to a trimer could occur with the leash occupying the same groove formed between monomers of the trimer as occupied in the post-fusion form of class II flavivirus E. Likewise, the stem of JMTV VP1 could occupy the same grove occupied by the stem of YFV E in the post-fusion configuration.

Immature virions of members of the *Flavivirus* genus of the *Flaviviridae* contain a precursor prM to the small membrane protein M [60]. The prM is cleaved by furin or by a furin-like protease during virus release to produce the mature M protein localized on the surface of flavivirus virions. Flavivirus prM/M contains two potential membrane spanning domains, and their functions may include shielding of internal cellular membranes from the fusion peptide of E [61]. E and M are likely present in equal molar amounts in the virion. Each dimer of E/M is associated with eight TM domains (Figure 4, one M is not shown). The E/M trimer would then have twelve TM domains. By comparison, the dimers of Jingmen flavi-like viruses would be associated with six TM in the dimers and nine in the trimer or fewer in the case of YGTV.

## 4. Discussion

Segment 2 of Jingmen flavi-like viruses encode either one or two proteins. The amino terminal end of VP1, the sole glycoprotein of certain Jingmen group viruses (JMTV, MGTV, AMTV, and others), bears sequence similarity to the N-terminus of VP1a, the longest of the two envelope proteins encoded by other Jingmen viruses (ALSV, YGTV and others); VP1b is similar to the C-termini of VP1. VP1, VP1a, and VP1b each have an amino terminal signal peptide. VP1 and VP1b have one or more TM domains. The VP1a of ALSV also has a TM domain, but the VP1a of YGTV lacks this feature. These observations suggest that segment 2 ALSV and YGTV was derived from segment 2 of a JMTV-like progenitor by mutational events that resulted in termination of the VP1a reading frame, followed by a shift in the reading frame and the insertion of a signal peptide for the VP1b portion of the gene.

Based on common features and computer algorithms, Gallaher and colleagues suggested [25] that the ectodomain of the transmembrane proteins of HIV-1 and other retroviruses fit the scaffold of the postfusion structure of influenza virus hemagglutinin 2 [62]. Common structural motifs, including extended α-helices and an N-terminal fusion peptide, were subsequently observed in the glycoproteins of filoviruses [24], arenaviruses [26], coronaviruses [63], and paramyxoviruses, now referred to collectively as class I VFPs. The envelope glycoproteins of flaviviruses, alphaviruses, and viruses formerly classified as members of the *Bunyavirdae*, which are now classified in separate families under the order *Bunyavirales*, form the structurally distinct class II VFPs [55,56]. Class II VFPs are characterized by a β-sheet structure interspersed with short α-helices. Rhabdoviruses, herpesviruses, certain baculoviruses, and members of the *Thogatovirus* genus of the *Myxoviridae* have class III VFPs [64,65,66]. While no similarity of any Jingmen group virus glycoprotein to other viral glycoprotein has been detected by common homology search programs, application of the methods of Gallaher predicted that Jingmen group virus glycoproteins have structure features of class II viral fusion proteins. These predicted features include an ectodomain consisting of β-sheets and short α-helices, a fusion peptide with interfacial hydrophobicity and a three-domain architecture. Confirmation of these predictions will require structural analyses using X-ray crystallography or cryoelectron microscopy.

Jingmen group virus glycoproteins contain PST-rich sequences, a feature that is characteristic of mucin-like domains. Computational analyses predict that many of the serine and threonine residues are modified by the addition of O-glycans. All Jingmen group VP1 or VP1a have a predicted mucin-like domain near the N-terminus. VP1a is distinguished from VP1 by the presence of an additional PST-rich region near the C-terminus of the protein, which is predicted to contain several O-linked glycans. Jingmen flavi-like viruses that encode a single glycoprotein have a stem structure consisting of two predicted α-helices that proceed the first TM domain. While computational models can predict potential glycosylation sites and protein secondary structures with high reliability, confirmation by chemical analysis, crystallography, or other methods is required to establish that the sites contain glycans or the predicted secondary structures.

The presence of glycans on viral surface proteins may shield the virion from recognition by the host’s immune system [67,68,69]. Some viral glycoproteins are glycosylated at relatively few sites, whereas others are densely glycosylated with N-linked and O-linked glycans. Members of the *Flavivirus* genus of the *Flaviviridae* typically have zero to two N-linked glycans [70,71,72]. HIV-1, Lassa virus, and coronaviruses are prominent examples of viruses whose glycoproteins have multiple N-linked glycans [73,74,75]. Filoviruses, pneumoviruses, metapneumoviruses, herpesviruses and nairoviruses encode glycoproteins with mucin-like domains [27,46,76]. In addition to versions with a TM anchor, filovirus, pneumovirus and metapneumovirus glycoproteins are expressed as a secreted forms involving alternative translation strategies [77,78]. It is not known whether or not frameshifting occurs in Jingmen flavi-like virus segment 2.

**Figure 7:**
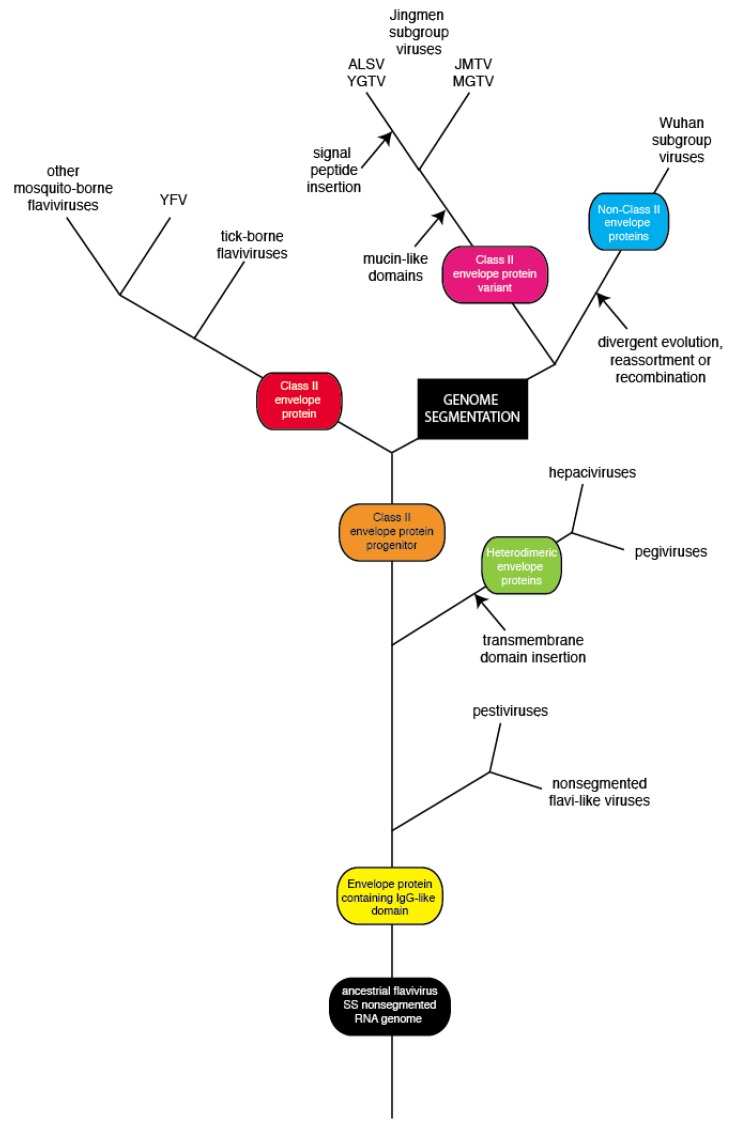
Possible evolution of Jingmen and Wuhan group viruses. The divergence of a progenitor of Jingmen and Wuhan group viruses from a progenitor of the members of the *Flavivirus* genus of the *Flaviviridae* occurred via genome segmentation. Following segmentation, Jingmen flavi-like viruses added one or more mucin-like domains to their glycoproteins. Wuhan group viruses replaced the class II envelope glycoprotein or a progenitor with distinct glycoproteins via recombination or divergent evolution.

The history of the *Flaviviridae* has been revised based on the discovery of the flavi-like viruses of invertebrates and other species [4]. Flavi-like viruses possess large unsegmented RNA genomes and occupy deep phylogenetic positions [4]. The *Flaviviridae* appear to have evolved from an ancestral flavi-like virus of invertebrates (Figure 7). The NS2B-NS3 and NS5 proteins of pestiviruses, one of four genera in the *Flaviviridae*, are most closely related among flaviviruses to the cognate proteins of unsegmented flavi-like viruses [4]. The structure of the pestivirus glycoprotein differs from the glycoproteins of other flaviviruses [79], which is consistent with a near-basal position of pestiviruses on *Flaviviridae* phylogenetic trees. Hepacivirus and pegivirus glycoproteins also differ from those of other flaviviruses [80]. The glycoprotein complex consists of two subunits, E1 and E2. However, E2 has an immunoglobulin-like (Ig-like) fold, which it shares with domain III of flavivirus E [81,82]. As suggested previously [80], an ancestral glycoprotein possessing an Ig-like fold could have been the progenitor of hepacivirus, pegivirus and subsequently other flavivirus glycoproteins.

The discovery of Jingmen viruses with four segment genomes encoding flavi-like NS2B-NS3 and NS5 proteins revealed an unexpected evolutionary link between unsegmented and segmented RNA viruses [7]. The NS2B-NS3 and NS5 proteins of Jingmen flavi-like viruses are more closely related to the cognate proteins of members of the *Flavivirus* genus than to those of the other genera (pestiviruses, hepaciviruses, or pegiviruses) suggesting that Jingmen viruses diverged from a common ancestral virus by the process of genome segmentation (Figure 7). The current studies suggest that flavivirus E and Jingmen flavi-like virus glycoproteins diverged from a common class II precursor glycoprotein. The divergence involved or was followed by the addition of mucin-like domains. A subgroup of Jingmen viruses, including ALSV and YGTV, diverged further by incorporating a frameshift in their segment 2 gene (Figure 7). The mutational events that resulted in the separation of the single ORF into two overlapping ORFs in segment 2 of ALSV and YGTV were also involved or were followed by events that generated a sequence encoding an additional mucin-like domain. Wuhan flavi-like viruses do not encode class II envelope proteins (Figure 7). There are several possibilities that could account for this difference, including divergent evolution or recombination/reassortment to acquire the gene for a distinct class of glycoprotein.

## 5. Conclusions

Jingmen flavi-like virus glycoproteins share a number of putative similarities with class II VFP but are predicted to diverge from known class II VFPs by the presence of one or more mucin-like domains. In the absence of structural determinations by X-ray crystallography or cryoelectron microscopy, models such as those proposed here can provide useful hypotheses to guide experimental strategies regarding this newly discovered group of viruses.

## Figures and Tables

**Table 1:** GenBank accession numbers used for structural analyses.

Virus	Segment 1	Segment 2	Segment 3	Segment 4
Alongshan virus	MN107156.1	MN107154.1	MN107155.1	MN107153.1
Yanggou tick virus	MH688529.1	MH688530.1	MH688531.1	MH688532.1
Jingmen tick virus	MN025512.1	MN025513.1	MN025514.1	MN025515.1
Mogiana tick virus	NC_034222.1	KY523073.1	NC_034223.1	KY523074.1
Wuhan cricket virus	KR902709.1	KR902710.1	NC_028395.1	NC_028402.1
